# Enhancing impact resistance and biodegradability of PHBV by melt blending with ENR

**DOI:** 10.1038/s41598-022-27246-z

**Published:** 2022-12-31

**Authors:** Napat Tomano, Orathai Boondamnoen, Chuanchom Aumnate, Pranut Potiyaraj

**Affiliations:** 1 Nanoscience and Technology Program, Graduate School, Chulalongkorn University, Bangkok, 10330 Thailand; 2 Department of Materials Science, Faculty of Science, Chulalongkorn University, Bangkok, 10330 Thailand; 3 Metallurgy and Materials Science Research Institute, Chulalongkorn University, Bangkok, 10330 Thailand; 4 Center of Excellence in Responsive Wearable Materials, Chulalongkorn University, Bangkok, 10330 Thailand

**Keywords:** Materials science, Biopolymers, Mechanical properties

## Abstract

This research aims to enhance the mechanical characteristics of poly(3-hydroxybutyrate-co-3-hydroxyvalerate) (PHBV) by using epoxidized natural rubber (ENR-25 and ENR-50) as a toughening agent and polybutadiene (PB) grafted with maleic anhydride (MA) (3 MA groups/chain) as a compatibilizer. The PHBV/ENR blends were mixed in 100/0, 90/10, 80/20, and 70/30 with PB-*g*-MA at 0, 5, and 10% (wt./wt.), using an internal mixer set to 175 °C with a rotor speed of 50 rpm. The findings indicated that at 70/30 PHBV/ENR composition, the impact strength of the blends with 25 and 50 epoxide contents were the greatest at 6.92 ± 0.35 J m^−1^ and 7.33 ± 1.19 J m^−1^, respectively, which are about two times greater than that of neat PHBV. Furthermore, the biodegradability of the PHBV/ENR blends was more substantial than that of neat PHBV, showing a mass reduction of approximately 40% and 45% for PHBV/ENR-25 and PHBV/ENR-50, respectively. In comparison, while the mass loss of PHBV was approximately 37% after three months of soil burial. The results indicate that ENR improves the toughness of the blends while simultaneously increasing PHBV degradation, which could pave the way for broadening PHBV for sustainability purposes.

## Introduction

In recent years, petroleum-based products have been one of the most worrying environmental issues since the causes cannot be regenerated, and they are resistant to biological, physical, and chemical degradation^1^. That has a significant role in the causes of environmental problems like global warming and the loss of a diverse range of organisms in the world's ecosystem and habitat^2^. Thus, bio-based materials have been developed to replace petroleum-based materials and are potentially utilized for commercial products^3^. Biodegradable plastics, the bio-based materials that could be sourced from nature and degraded spontaneously by microbial activity, such as poly(3-hydroxybutyrate), (PHB) and poly(3-hydroxybutyrate-co-3-hydroxyvalerate), (PHBV), which are also known as biodegradable aliphatic polyester in a group of poly(3-hydroxyalkanoates) (PHAs)^4,5^. PHAs can be produced through the fermentation process of bacteria such as *Alcaligenis euterophus*, *Bacillus*, and *Pseudomonas* in an oxygen and carbon dioxide regulated environment^6–9^. Interestingly, it rapidly decomposes into water and carbon dioxide^10^.

PHBV is a promising bioplastic used in various biomedical applications, such as drug-delivery carriers, scaffolds, bone repair, orthopedic devices, and the packaging industry. Also, PHBV is generally tougher than PHB because of its chemical functionalization. However, one of the critical drawbacks is its thermal instability, which could bring about a narrow processing window. The degradation of PHBV could be rapidly accelerated during conventional processing methods such as melt extrusion and injection molding, resulting in restricted PHBV usage^11^. Moreover, the high crystallinity of up to 70% and slow nucleation rate make PHBV brittle, leading to poor mechanical characteristics^12^. Thus, many studies have been undertaken to explore strategies to enhance physical and functional properties, which could strengthen the possibility of PHBV as sustainable packaging material. Blending PHBV with other polymers is a proposed solution to reduce crystallinity and improve toughness^13,14^. Besides, it is not only for enhancing the mechanical performance, but biodegradability is also preserved. Many approaches for toughening PHBV have been suggested, such as thermal treatment modification via annealing to decrease crystallinity^15^ and blending with other flexible polymers, such as PBS^16^. However, the compatibility between PHBV and the blending polymer is a critical concern; for example, the compatibility between PHBV and PBS was poor due to relatively large particle size and weak interfacial adhesion in their blends. The same phenomenon was observed for PHBV/Polybutylene adipate terephthalate (PBAT) blends^17^. Moreover, it was reported that the addition of PBAT led to a decrease in crystallinity consistently with increasing PBAT composition in the blends.

Among various polymers, natural rubber (NR) is a promising sustainable toughening agent from renewable natural resources with high molecular weight, low glass transition temperature, flexibility, and ductility^18^. Epoxidized natural rubber (ENR) is a chemically modified natural rubber from epoxidation process. Herein, the double bonds of isoprene units are replaced with epoxide groups^19^. The degree of epoxidation may be effectively controlled by time processing, with 25 and 50 mol% almost being utilized. Commercially available ENR with 25 and 50 mol% epoxy contents are named ENR-25 and ENR-50, respectively. The epoxide group content is projected to significantly impact the reactivity to blending with polymers than natural rubber^20^. ENR is more compatible with polymers containing polar functional groups, such as PHBV. The effect of ENR and NR as toughening agents for PHB was studied. It was reported that the addition of 40% ENR increased the toughness of the PHB/ENR blend, while the PHB/NR blend did not show an improved toughness. This may be attributed to the produced unbonded rubber phase in PHB/NR blends. Also, it was reported that the blending of PHBV/ENR exhibits good compatibility with further improved toughness compared to the PHBV/NR blend^21,22^.

In addition, adding compatibilizers is one of the effective attempts to improve the compatibility of blending PHBV with other polymers. It could prevent coalescence and enhance interfacial adhesion, thus reducing the phase separation between blending compositions^23^. Maleated polybutadiene is a compatibilizer applied for PHB/ENR blends. With its high grafting and low molecular weight, the interfacial adhesion between the PHB and ENR could be achieved, thus improving the toughness characteristic of the blends^22,24,25^. However, the effect of adding compatibilizers on improving the properties of PHBV/ENR blends has rarely been reported.

This study aims to improve the toughness of PHBV by melt-blending with different epoxidized rubbers, ENR-25, and ENR-50, using an internal mixer. Herein, the effect of epoxide contents and compatibilizer (PB-g-MA) on improving the mechanical properties of the PHBV/ENR blends was investigated in terms of notched impact strength, tensile properties, and flexural properties. The thermal behaviors of the blends were examined by thermogravimetric analysis (TGA) and differential scanning calorimetry (DSC), respectively. The crystal structure was monitored by x-ray Diffraction (XRD) analysis. Also, the blend morphology was studied by scanning electron microscopy (SEM). Moreover, the biodegradability of the PHBV/ENR blends was also investigated by burying them in the soil for three months.

## Results and discussion

**Figure 1:**
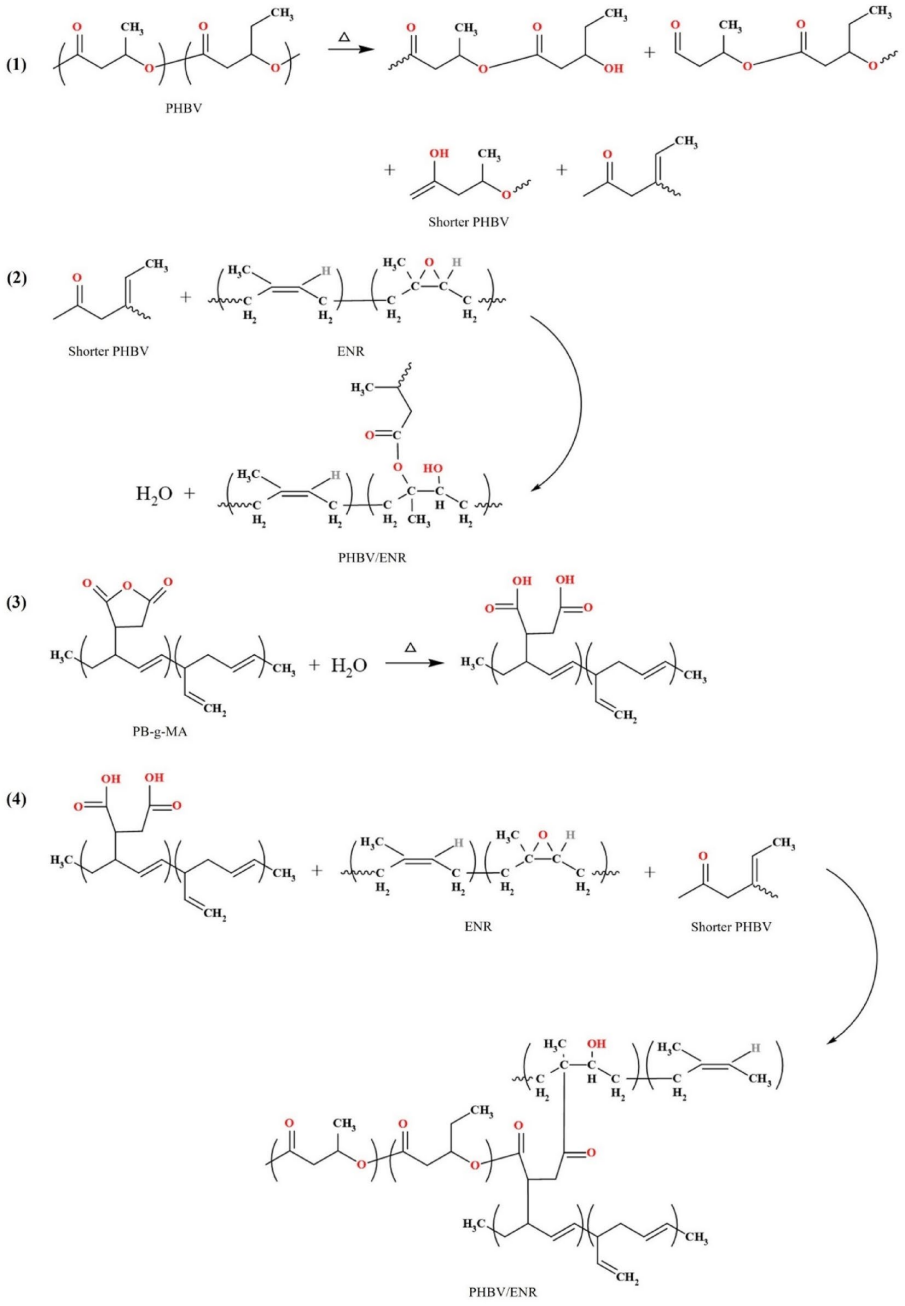
Schematic illustration of the proposed reactions between PHBV, ENR, and PB-g-MA.

Figure 1 presents the probable reactions during the melt-blending process with and without a compatibilizer. The reaction begins with a random thermal scission of long-chain PHBV molecules to shorter chains containing carboxyl ends^15,26^, as shown in Eq. (1). In the presence of ENR, the epoxide ring in the vicinity of the newly generated carboxyl groups was open as Eq. (2). It is important to note that the hydroxyl group is thus produced and reactive towards the carboxyl and epoxide groups. When the reaction was completed, water was found as a byproduct. The potential reaction pathway of the blends using PB-g-MA as a compatibilizer begins with the ring-opening of maleic anhydride by heat reacting with water to create hydroxyl groups, as shown in (3)^27^. Finally, the obtained product from the reaction as Eq. (3) reacts at the oxirane ring of ENR and the carboxyl of PHBV, as shown in Eq. (4)^28^.

### Thermal stability

**Figure 2:**
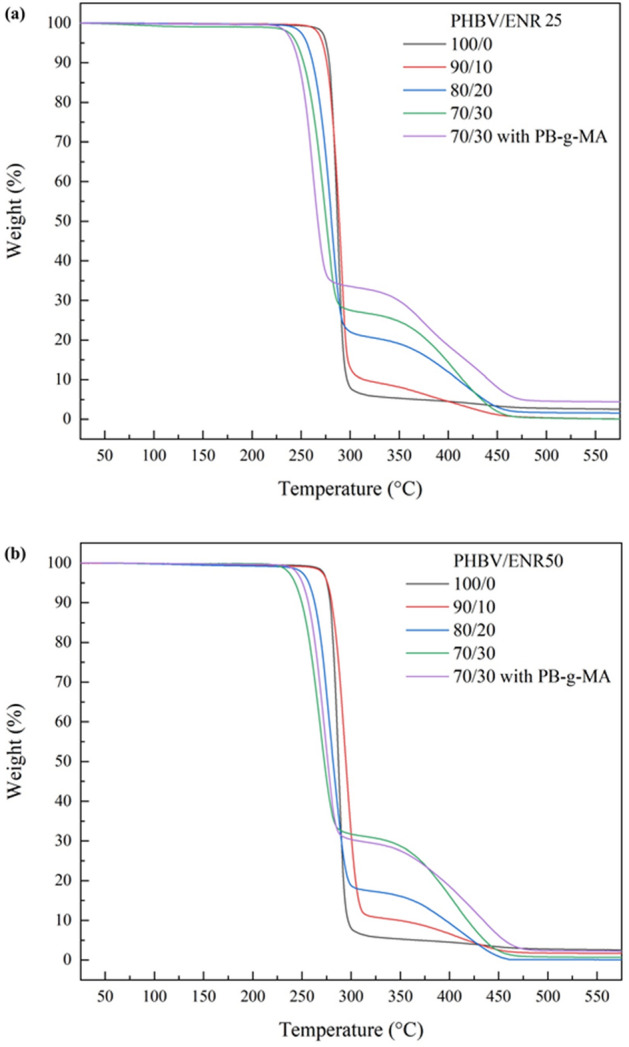
The TGA thermogram of neat PHBV (100/0) and PHBV/ENR blends with and without compatibilizer (PB-g-MA), (**a**) PHBV/ENR-25 blends, and (**b**) PHBV/ENR-50 blends.

Figure 2 presents the TGA thermogram of the neat PHBV (100/0), and PHBV/ENR blends with and without compatibilizer (PB-g-MA). It was observed that PHBV/ENR blends were degraded in two stages, revealing the differences in the thermal stability of PHBV and ENR. The first stage demonstrated PHBV decomposition between 254.3 and 304.0 °C^26^, whereas the second stage, between 272.8 and 460.0 °C and between 280.8 and 460.0 °C^29^, revealed the decomposition for ENR-25 and ENR-50, respectively. During the melt-blending process, the thermal could accelerate random chain scission of PHBV, creating olefinic compound, carboxylic acid, crotonic acid, and oligomer^30^. PHBV could also depolymerize to decrease molecular weight by acid from random chain scission reaction. The higher the ENR content, the more significant deterioration, which may be attributed to the more heat dissipation of ENR^31^. As a result, the thermal stability of the blends tended to be reduced with the ENR loading. On the other hand, the degradation temperature of the ENR counterpart was enhanced in proportion to higher ENR concentration, attributing to the interaction with PHBV surrounding the ENR phase retarding ENR degradation. Nevertheless, less thermal stability was observed for the blends with 5% PB-g-MA. This may be ascribed to the higher compatibility level between PHBV and ENR, which resulted in a smaller phase distribution of ENR, thus inducing more heat dissipation to PHBV^32,33^. For all blend compositions, the blends with ENR-50 exhibited slightly higher thermal stability than those with ENR-25 because of higher epoxidation content^34^.

### Thermal properties

**Figure 3:**
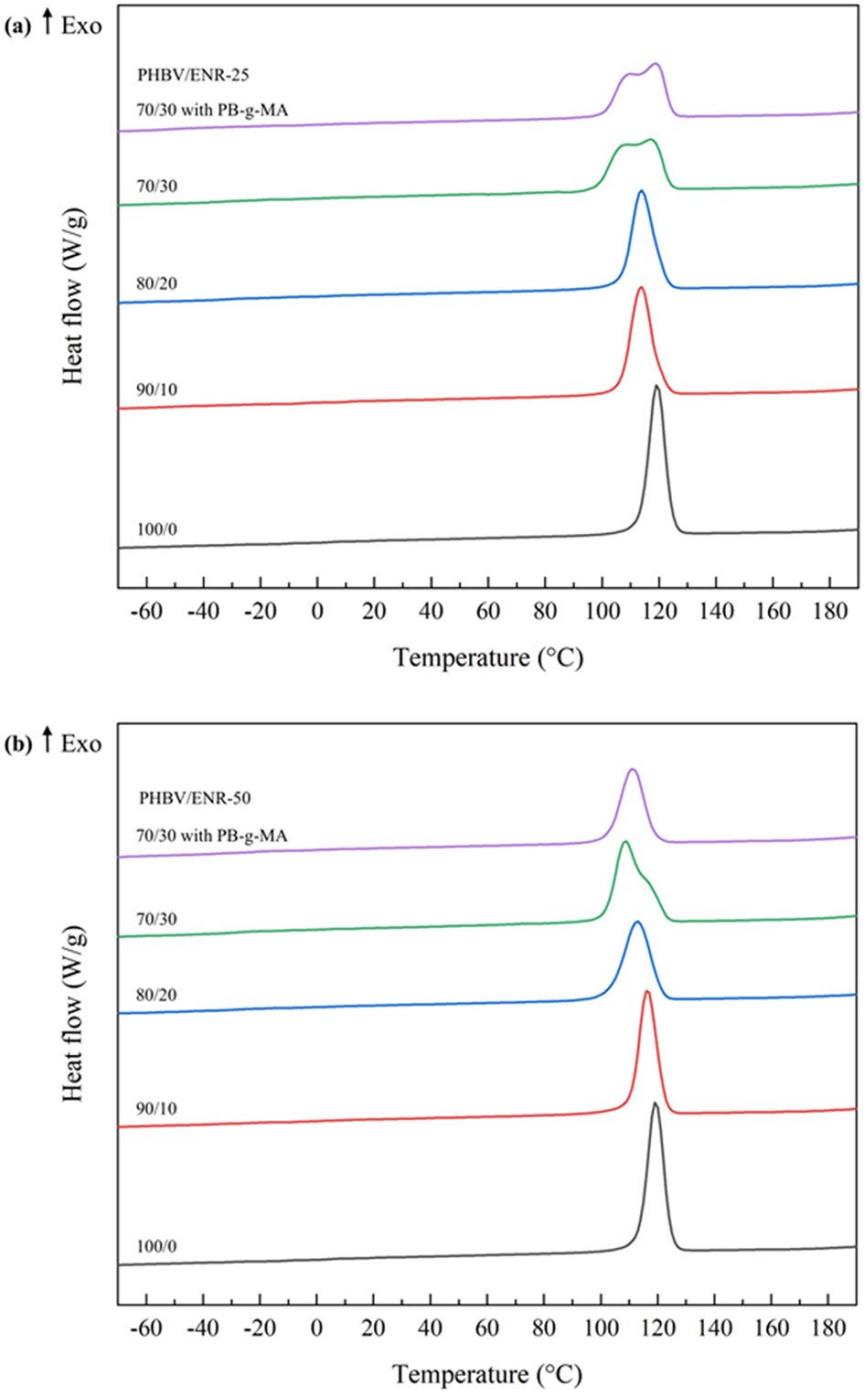
The second heat DSC thermogram of PHBV (100/0) and PHBV/ENR blends with and without compatibilizer (PB-g-MA), (**a**) PHBV/ENR-25 blends and (**b**) PHBV/ENR-50 blends.

Figure 3 displays the second heat DSC thermogram of PHBV and its blends. The glass transition temperature (T_g_) for all PHBV/ENR blends was not observed due to the high crystallinity of PHBV. A single melting temperature (T_m_) peak was presented at 171.1 °C for the neat PHBV (100/0). For all blends, the broader T_m_ peaks ranged from 164.0 to 172.4 °C. Incorporating ENR seems to have significant effects on the crystallization behavior of the blends. The increase in the ENR loading, the lower the T_m_. Moreover, increasing the ENR loadings resulted in a broader melting peak and lower degree of crystallinity (X_c_), particularly for blends with 30 w% of ENR content (for both ENR-25 (a) and ENR-50 (b)). The transition temperatures and the parameters obtained from the DSC measurement of PHBV/ENR blends are demonstrated in Table 1. The melting and crystallization behaviors of polymer blends were significant in deciding mechanical properties^35^. The decrease in crystallization temperatures (T_c_) and the degree of crystallization (X_c_) may be associated with the development of imperfect crystallites. The possibility of the ENR phase migrating into the inter- and intra-spherulitic regions of crystalline PHBV restricted the crystal development from its preferred crystalline morphology^36,37^. Moreover, the compatibility of PHBV/ENR blends with a compatibilizer (5wt% PB-g-MA) resulted in a lower interfacial contact area between the PHBV and ENR phases. As a result, the polymer networks could be generated, thus inhibiting PHBV crystallization^38–40^.

**Table 1:** DSC parameters of PHBV and PHBV/ENR blends.

Samples	T_c_ (°C)	T_m_ (°C)	ΔH_c_ (J g^-1^)	ΔH_m_ (J g^-1^)	X_c_ (%)
PHBV (100/0)	119.3	171.1	92.0	91.6	84.0
**PHBV/ENR-25**					
90/10	113.7	171.4	85.1	95.6	87.7
80/20	113.8	167.6	80.7	86.6	79.4
70/30	117.0	167.6	77.3	86.7	79.6
70/30 with PB-g-MA	118.8	168.1	74.3	77.8	71.4
**PHBV/ENR-50**					
90/10	116.4	172.4	84.5	91.6	84.1
80/20	112.8	167.6	75.9	82.3	69.7
70/30	108.7	164.0	83.9	91.6	84.0
70/30 with PB-g-MA	111.2	169.8	65.2	71.7	65.7

### Crystal structure

**Figure 4:**
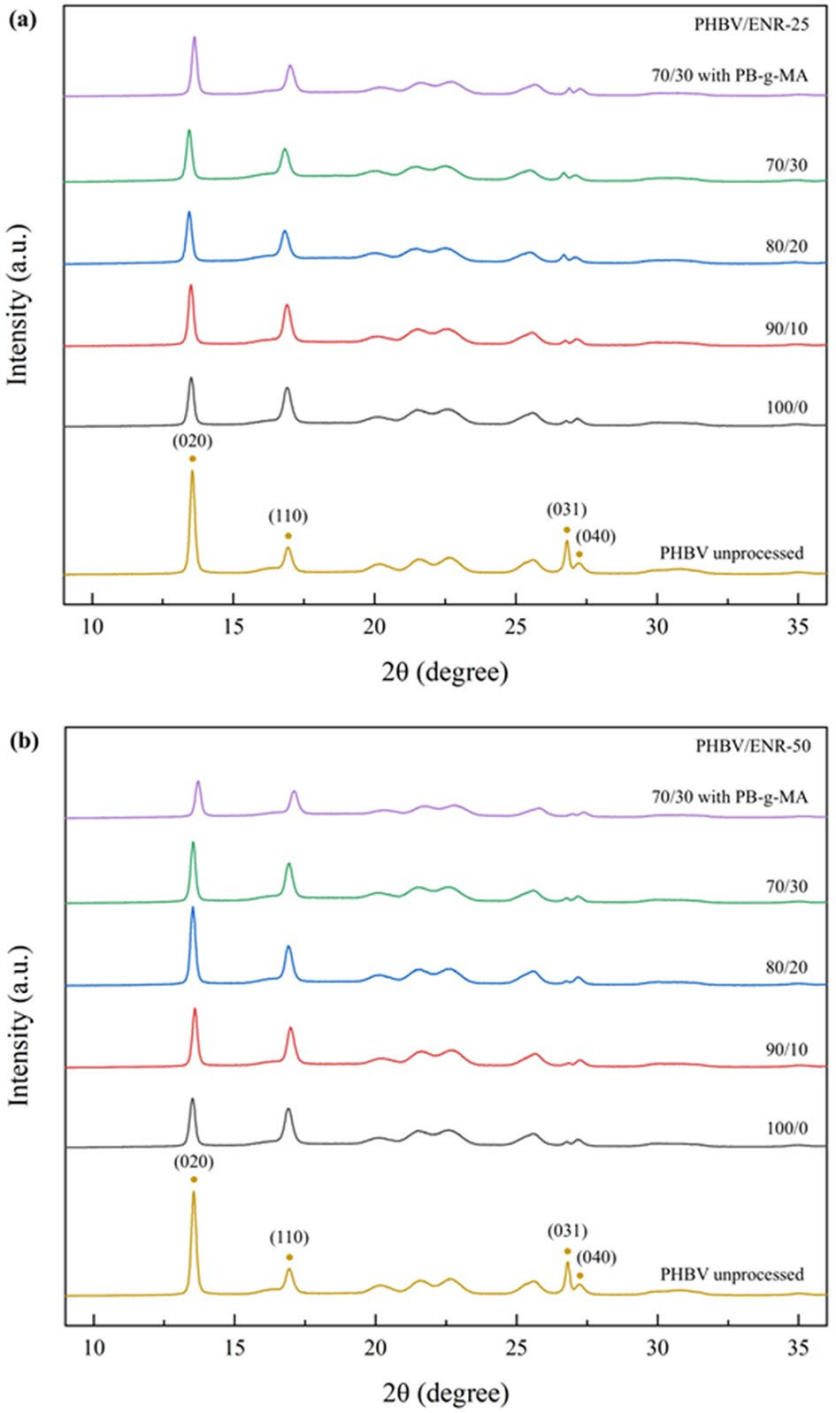
XRD diffractograms of the neat PHBV (PHBV unprocessed) and PHBV/ENR blends (**a**) PHBV/ENR-25 blends and (**b**) PHBV/ENR-50 blends.

The XRD diffractograms of PHBV and all blends are shown in Fig. 4. The orthorhombic crystal structure was shown by reflection peaks at 13.25°, 16.63°, 25.17°, and 26.52°, which correspond to the (020), (110), (031), and (040) planes^41^. Nonetheless, the degree of crystallinity of the blends was considerably reduced, confirming the DSC results. No new peak was detected in the XRD patterns for all blends, showing no new crystalline phase occurred^42^. Compared with the neat PHBV (PHBV unprocessed), the planes of (020), (110), (031), and (040) were shifted to higher angles and broader for all blends. This could be described as the crystal growth of PHBV in the blends being restricted by the dispersed ENR phase, suggesting that the melt-blending with ENR significantly reduced the crystallinity of the PHBV^43^. Similar results were found for either blending with ENR-25 or ENR-50.

### Mechanical properties

The mechanical properties of polymer blends are directly governed by their compatibility between blending compositions^44^. This section discussed the effect of PB-g-MA loadings, 5wt%, and 10wt%, on the blends' mechanical properties. Tables 2 and 3 present the mechanical properties of the PHBV and blends with ENR-25 and ENR50, respectively.

**Table 2:** Mechanical properties of the PHBV and PHBV/ENR-25 blends.

Samples	Compatibilizer (%)	Impact strength (J/m)	Tensile properties			Flexural modulus (MPa)	Strain (%)
			Tensile strength (MPa)	Elongation at break (%)	Young’s modulus (MPa)		
100 PHBV	–	3.30 ± 0.66	28.34 ± 3.37	6.65 ± 0.73	868.29 ± 149.77	3,020.89 ± 178.00	4.14 ± 0.81
**PHBV**/**ENR-25**							
90/10	–	4.72 ± 0.10	21.98 ± 2.44	5.51 ± 0.62	851.79 ± 36.00	2,287.90 ± 59.36	6.72 ± 0.52
80/20	–	5.51 ± 0.56	13.85 ± 1.93	2.70 ± 0.18	689.78 ± 39.39	1,775.94 ± 65.37	7.10 ± 0.66
70/30	–	6.76 ± 0.43	5.60 ± 1.09	5.47 ± 0.66	343.59 ± 76.89	671.25 ± 47.03	8.09 ± 1.19
**PHBV**/**ENR-25**/**5%PB-g-MA**							
90/10	5	3.30 ± 0.86	25.83 ± 1.43	5.35 ± 0.49	802.71 ± 53.78	2,655.55 ± 96.24	5.24 ± 0.47
80/20	5	4.72 ± 0.97	15.01 ± 1.03	3.56 ± 0.12	655.82 ± 24.41	1,760.64 ± 61.46	6.09 ± 0.66
70/30	5	6.92 ± 0.35	4.70 ± 0.60	3.36 ± 0.22	315.90 ± 4.52	1,240.33 ± 192.55	7.63 ± 0.89
**PHBV**/**ENR-25**/**10% PB-g-MA**							
90/10	10	1.57 ± 0.10	28.00 ± 2.92	6.02 ± 1.21	819.74 ± 75.96	2,512.57 ± 91.43	4.81 ± 0.26
80/20	10	2.99 ± 0.66	14.06 ± 1.98	2.88 ± 0.41	637.43 ± 52.53	1,837.75 ± 90.52	5.32 ± 0.46
70/30	10	4.56 ± 0.66	5.67 ± 0.60	2.63 ± 0.61	361.97 ± 53.15	825.72 ± 52.49	5.78 ± 0.72

**Table 3:** Mechanical properties of the PHBV and PHBV/ENR-50 blends.

Samples	Compatibilizer (%)	Impact strength (J/m)	Tensile properties			Flexural modulus (MPa)	Strain (%)
			Tensile strength (MPa)	Elongation at break (%)	Young’s modulus (MPa)		
100 PHBV	–	3.30 ± 0.66	28.34 ± 3.37	6.65 ± 0.73	868.29 ± 149.77	3,020.89 ± 178.00	4.14 ± 0.81
**PHBV/ENR-50**							
90/10	–	5.04 ± 0.43	22.64 ± 2.41	6.57 ± 0.61	751.70 ± 43.87	2,670.14 ± 81.03	6.09 ± 0.63
80/20	–	5.19 ± 0.43	12.58 ± 1.96	2.95 ± 0.66	652.04 ± 76.78	1,769.67 ± 58.47	6.46 ± 0.58
70/30	–	5.66 ± 1.17	8.24 ± 1.65	2.31 ± 0.68	534.33 ± 6.02	1,285.12 ± 92.16	6.81 ± 0.92
**PHBV/ENR-50/5%PB-g-MA**							
90/10	5	3.46 ± 0.71	22.46 ± 2.59	6.14 ± 1.20	712.49 ± 73.36	2,733.01 ± 19.17	5.08 ± 0.22
80/20	5	4.13 ± 0.98	17.40 ± 1.40	4.39 ± 0.62	655.65 ± 73.88	1,813.33 ± 49.84	6.36 ± 1.01
70/30	5	7.33 ± 1.19	5.55 ± 1.18	3.98 ± 1.35	328.41 ± 32.40	1,236.34 ± 143.46	8.29 ± 0.61
**PHBV**/**ENR-50**/**10% PB-g-MA**							
90/10	10	2.20 ± 0.35	27.28 ± 2.16	5.36 ± 0.63	844.05 ± 37.76	2,390.16 ± 95.70	5.45 ± 0.72
80/20	10	3.46 ± 0.43	19.29 ± 1.57	4.50 ± 0.35	644.01 ± 66.95	1,763.56 ± 52.36	6.99 ± 0.37
70/30	10	4.72 ± 1.11	10.59 ± 0.93	3.43 ± 0.58	407.06 ± 75.13	1,186.28 ± 90.60	7.32 ± 0.44

### Tensile properties

**Figure 5:**
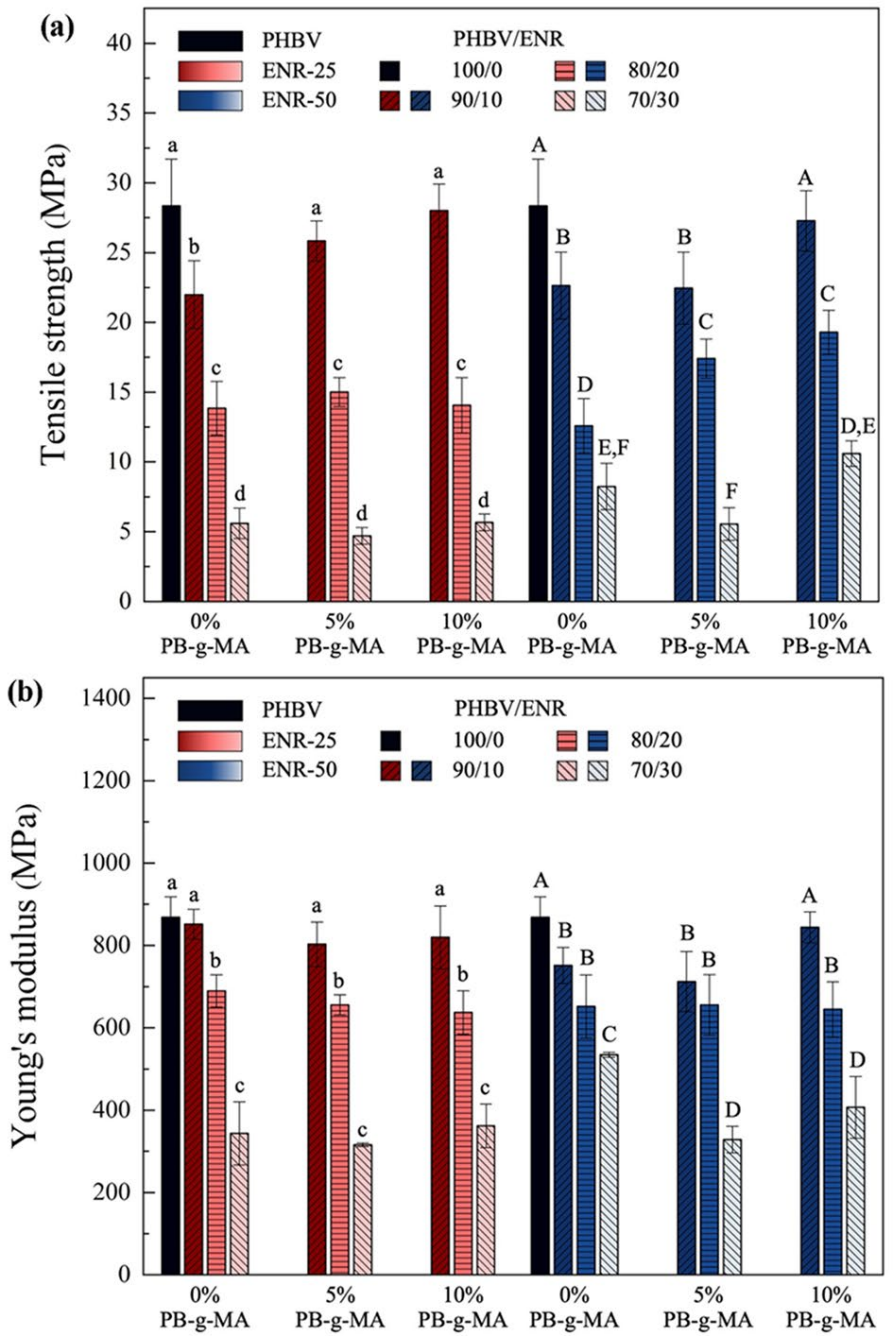
The tensile properties of PHBV/ENR blends (**a**) Tensile strength and (**b**) Young’s modulus. Data are presented as mean ± SD. Groups designated with different letters are significantly different using One-Way ANOVA with Duncan’s multiple range post hoc (at *p* < 0.05, n = 3).

The ENR diminished tensile strength and Young’s modulus of the blends because of the softening effect of the ENRs, and voids formation in their structures^45^ due to the compatibility between PHBV and ENR. During the melt blending process, PHBV chains were broken into shorter chains before reacting with the compatibilizer and ENR (see Fig. 1), reducing the crystallinity and directly affecting the tensile characteristics. As shown in Fig. 5, for the PHBV/ENR-25 blends, the tensile strength significantly decreases with increasing the ENR-25 content due to the rubber's low modulus and strength characteristics^46,47^. The addition of a compatibilizer, PB-g-MA, significantly improved the tensile strength of the blends with 10wt% ENR-25 content. However, for higher ENR-25 contents, 20 and 30wt%, the presence of PB-g-MA has no significant effect on improving the tensile strength of the blends. For PHBV/ENR-50 blends, the presence of compatibilizer significantly improved the tensile strength of all blends. A significant tensile strength improvement was observed for the blends with 10wt% PB-g-MA addition. It is worth noting that the higher the PB-g-MA content, the higher the tensile strength achieved due to the compatibility between PHBV and ENR induced by the content of epoxidation. A similar phenomenon was also observed for Young’s modulus.

### Flexural properties

**Figure 6:**
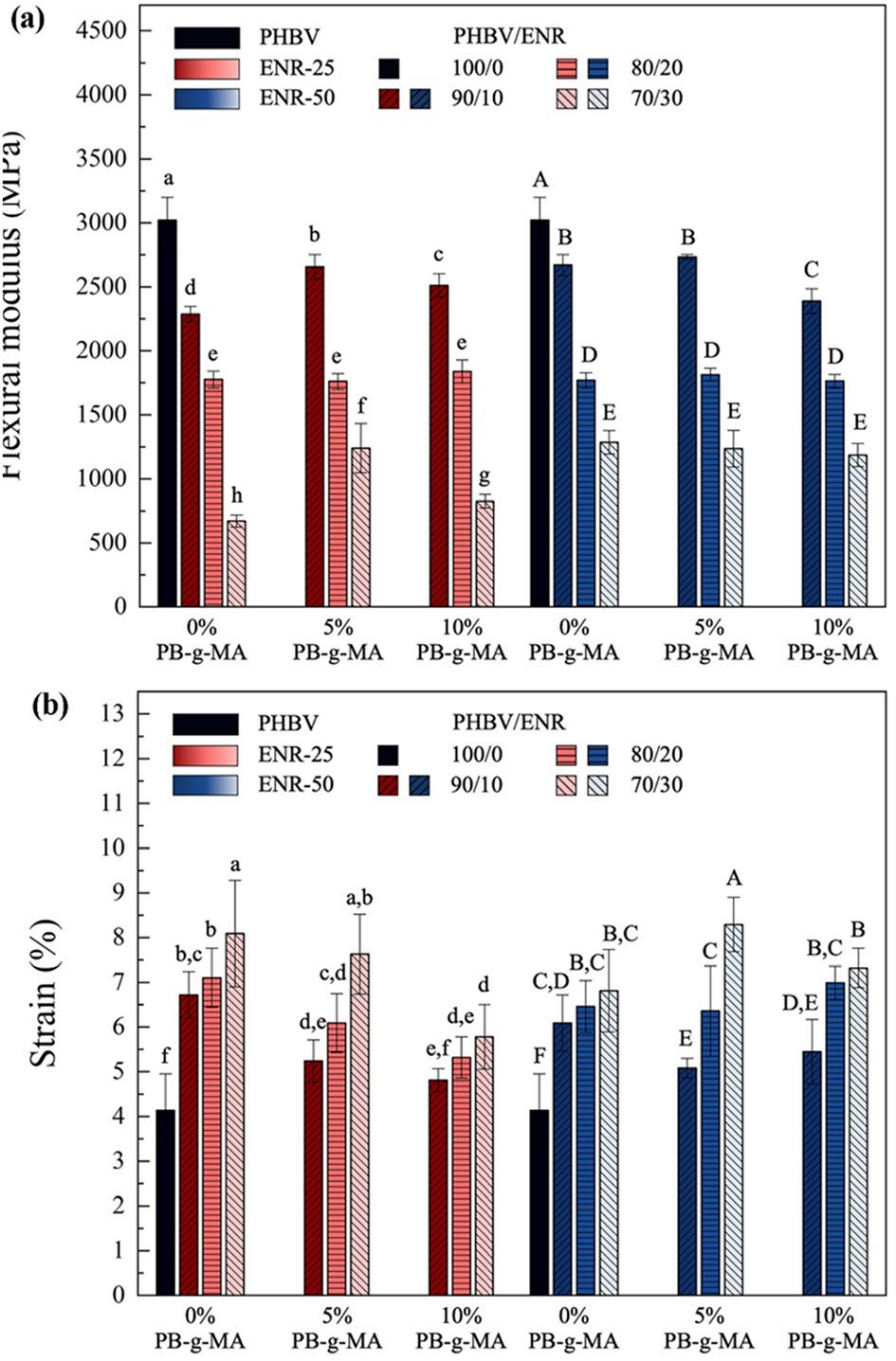
The flexural properties of PHBV/ENR blends (**a**) Flexural modulus and (**b**) Strain at break. Data are presented as mean ± SD. Groups designated with different letters are significantly different using One-Way ANOVA with Duncan’s multiple range post hoc (at *p* < 0.05, n = 3).

Good compatibility of PHBV and ENRs, resulting from physical or chemical interactions, is crucial to achieving the desired mechanical properties. The flexural modulus of the blends significantly decreased with increasing ENR loading. However, the strain at break increased, as shown in Fig. 6. This is due to the low modulus and flexible nature of ENR^48,49^ in the blends. As demonstrated in Fig. 1, the part of the PHBV chain could be cross-linked with the ENR chain using PB-g-MA as a compatibilizer. The separation between PHBV and ENR phases may cause an initial crack propagation that governs the mechanical performance of the blends. The compatibilizer, PB-g-MA, could induce the chemical interaction between PHBV and ENR, thus increasing the strain at break. For PHBV/ENR-50, the addition of PB-g-MA showed a significant improvement in the strain at break, particularly in the blends with ENR contents larger than 10wt%. The most promising mechanical properties were obtained from PHBV/ENR-50 (70/30) with 5wt% PB-g-MA.

### Impact properties

**Figure 7:**
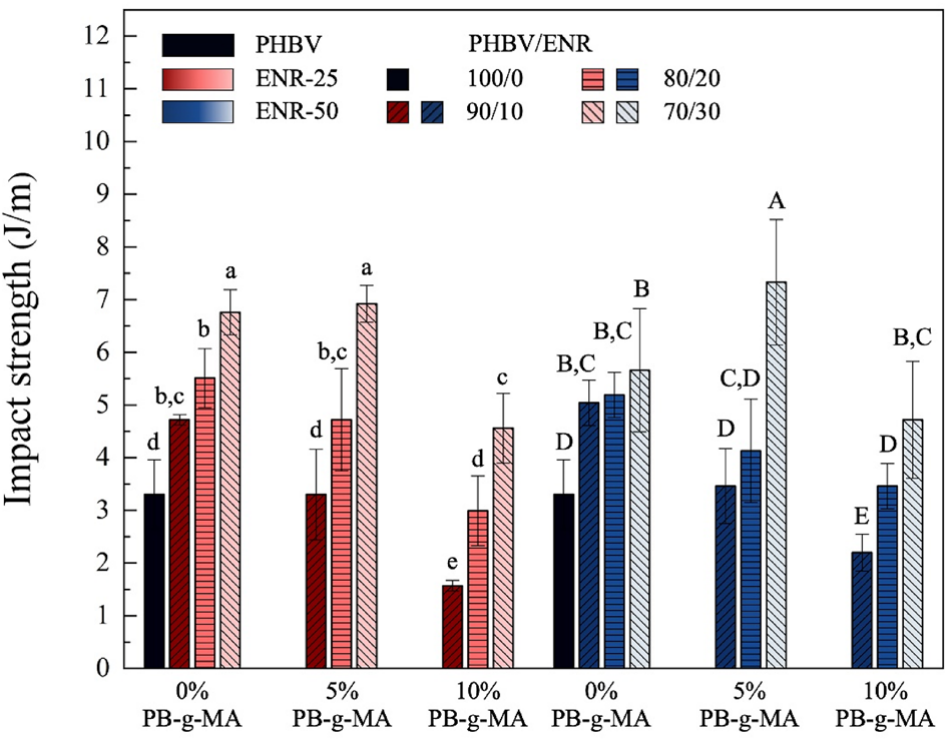
The impact strength of PHBV/ENR blends. Data are presented as mean ± SD. Groups designated with different letters are significantly different using One-Way ANOVA with Duncan’s multiple range post hoc (at *p* < 0.05, n = 3).

Notched impact strength is utilized to evaluate materials' toughness (impact resistance)^45^. Figure 7 presents the impact strength of the neat PHBV and PHBV/ENR blends with and without PB-g-MA addition. The impact strength of the neat PHBV is 3.33 ± 0.66 J m^-1^, exhibiting the brittleness characteristic due to its high crystallinity. However, the impact strength was significantly increased with ENR incorporation ranging from 10 to 30wt% because of the elastomeric behavior of ENR^20^. The impact strength increased with the ENR loading. At a small ENR concentration (10 wt%), the ENR phase was too far from each other to retain enough stress concentration to improve toughness^50^. However, when the ENR content reached 30wt%, the ENR fragmented into small phases and was distributed well in PHBV, yielding toughness enhancement^51^. The smaller size of ENR enhances the impact strength due to good interfacial adhesion (although the average particle diameter was larger than 1 μm). These results confirmed that the ENR could be used as an impact modifier or toughening agent for PHBV.

The addition of compatibilizer (PB-g-MA) significantly improved the impact strength, particularly at 5wt% loading. The blends with 30 wt% ENR-50 and 5 wt% PB-g-MA contents showed the highest toughness modification. It should be noted that the more considerable amount of PB-g-MA (10 wt%) seems to give a worse result, which may be due to the exceeded amount of PB-g-MA causing the interaction between maleic anhydride of PB-g-MA, carboxyl of PHBV, and epoxide of ENR instead of creating the interfacial adhesion between PHBV and ENR phases^15,27^. The 70/30 PHBV/ENR-50 blend with 5 wt% PB-g-MA exhibited the most remarkable impact strength improvement. Different epoxide groups in natural rubber do not impact on toughness modification in various compositions. However, at 70/30 PHBV/ENR blends, the epoxide content seemed to impact the blends due to the good dispersion of rubber in the PHBV phase. In contrast, in other compositions, 10wt% and 20wt% ENR, the epoxide content had no significant effect on the toughness. The obtained results could be caused by the higher epoxide ring content that could promote PHBV chain scissions. To conclude, the PHBV/ENR blends with PB-g-MA addition improved mechanical attributes such as notched impact strength and strain at break, which could represent the successful approach for toughening PHBV.

### Morphology

**Figure 8:**
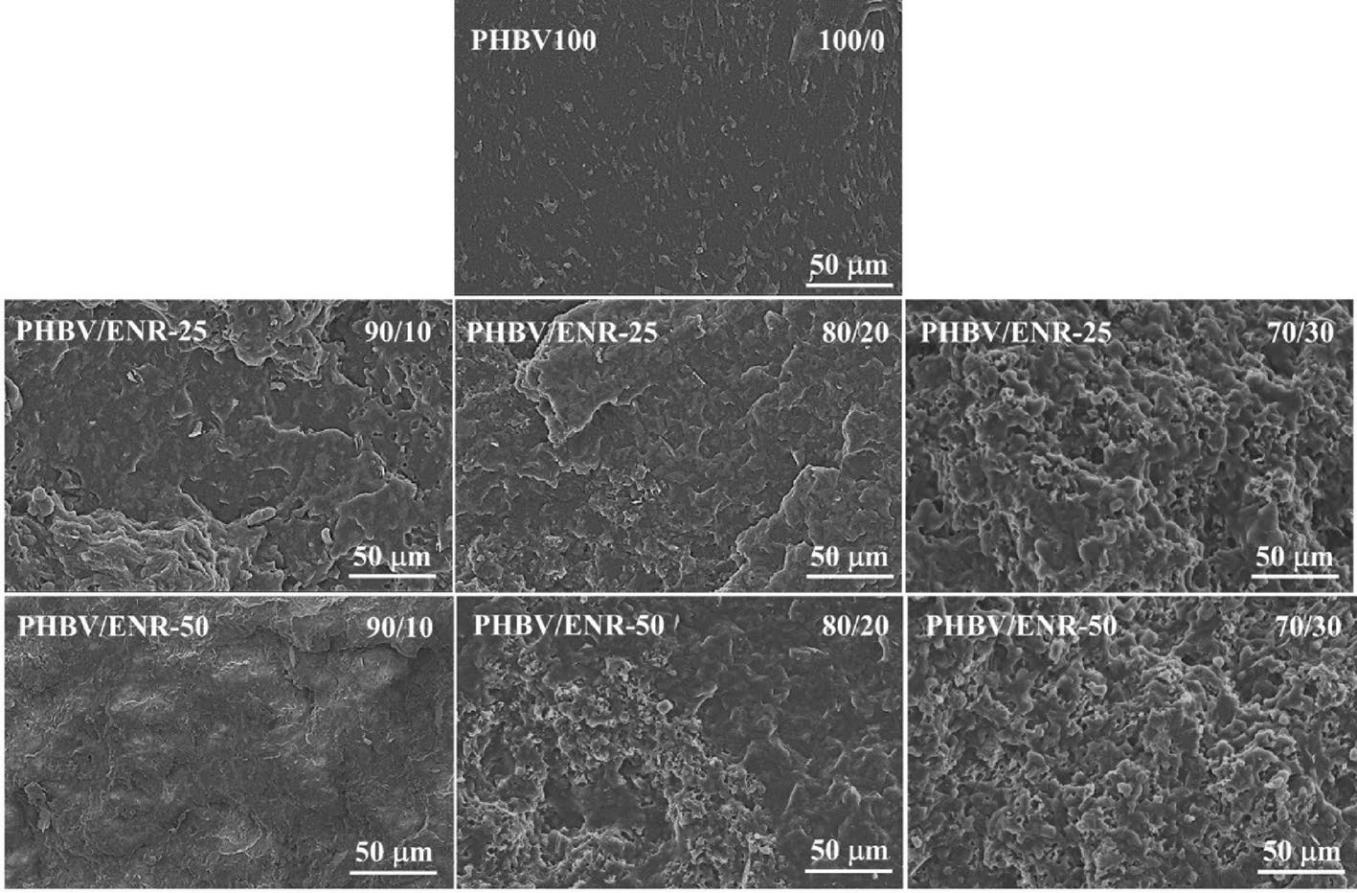
SEM micrographs of fractured surfaces of the neat PHBV, PHBV/ENR-25 blends, and PHBV/ENR-50 blends with the blending ratio of 90/10, 80/20, and 70/30.

**Figure 9:**
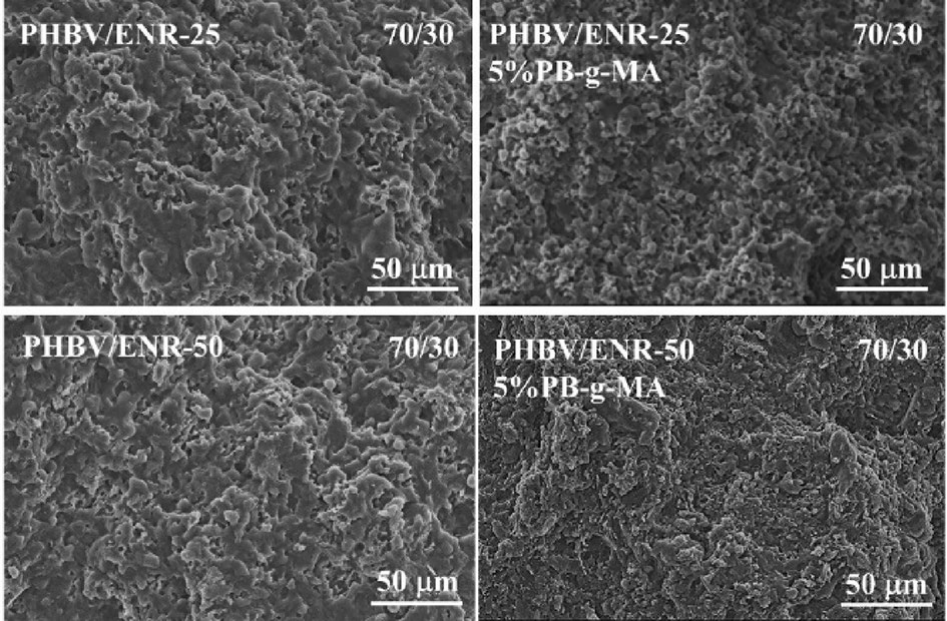
SEM micrographs of fractured surfaces of PHBV/ENR-25 and PHBV/ENR-50 blends with and without PB-g-MA.

The morphology of the neat PHBV and PHBV/ENR blends was anticipated from their mechanical characteristics. As shown in Fig. 8, the fracture surface of the neat PHBV was quietly smooth, characteristic of brittle materials^52^, and lacked bonding due to the high crystallinity properties^22,53^. The PHBV/ENR blends showed rough fracture surfaces with the presence of ductility materials^54,55^^.^ At small ENR contents (10–20 wt%), the fractured surfaces showed good dispersion and adhesion of ENR; although it is not possible to distinguish the PHBV phase and ENR phase, the obscure phase boundary could be confirmed, and no pulling out of ENR phase for the blends containing 10–20 wt% of ENR. At larger ENR loading (30 wt%), the packed and folded morphology was observed with the ENR phase dispersed across the PHBV matrix. Also, the addition of 5 wt% PB-g-MA led to a smaller size of ENR dispersed in the PHBV matrix, as shown in Fig. 9. The 70/30 PHBV/ENR blends with and without 5 wt% PB-g-MA exhibited ductile fracture, supporting an extensive PHBV/ENR interacting network^56^.

### Biodegradable properties (soil burial test)

**Figure 10:**
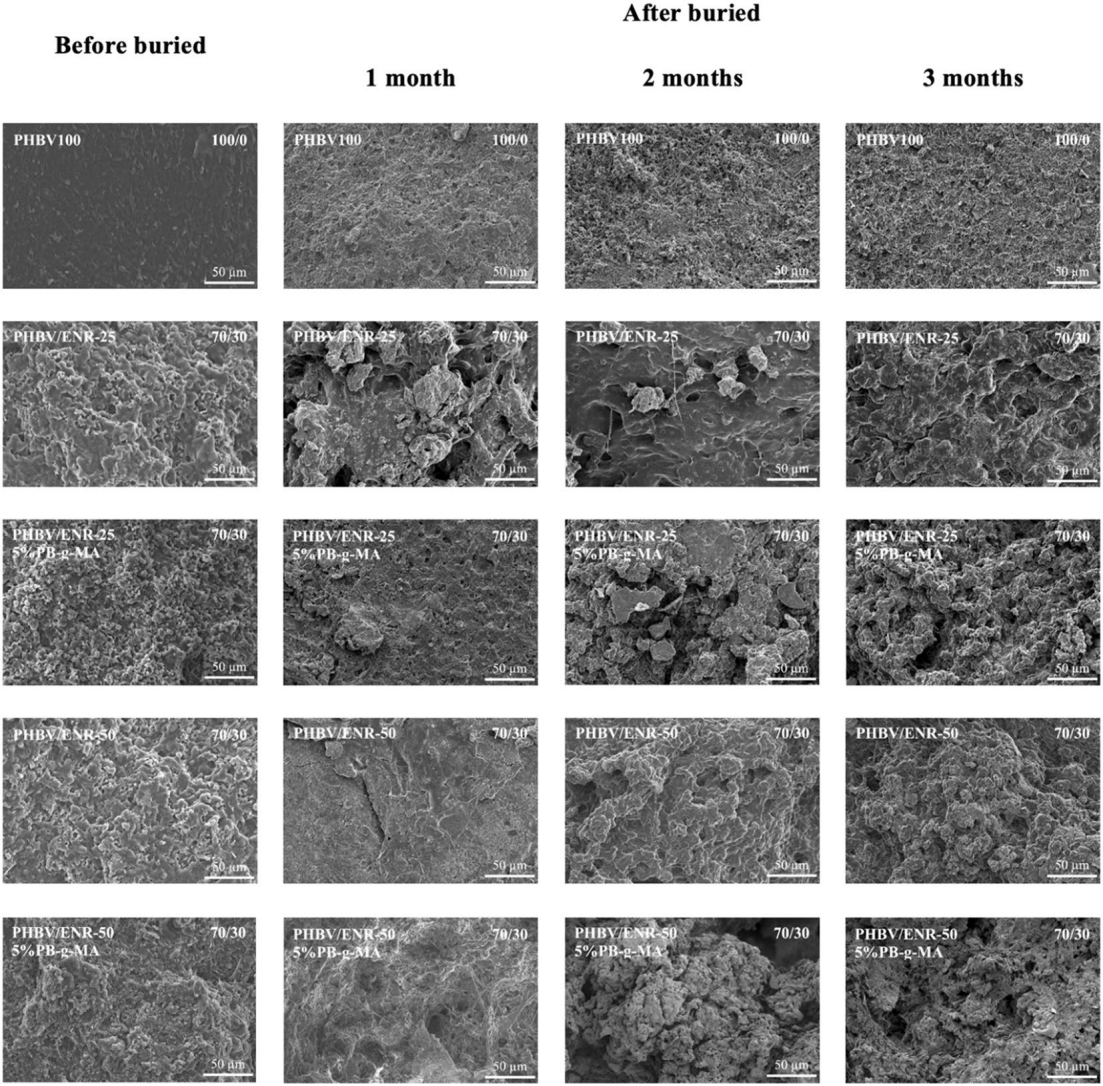
Representative SEM images of the neat PHBV, 70/30 PHBV/ENR-25 blends, and 70/30 PHBV/ENR-50 blends, with and without 5wt% PB-g-MA.

Figure 10 presents the surface morphology of the neat PHBV (100 PHBV) and 70/30 PHBV/ENR blends with and without 5wt% PB-g-MA before being buried (0 months) and after buried (1, 2, and 3 months) in the compost soil^57^. The neat PHBV exhibited a relatively smooth and clear surface before burial. However, after being buried for 1, 2, and 3 months in the compost soil, the samples showed different levels of degradation from the enzymatic action by living microorganisms in the compost soil.

As a result, a porous surface was developed and broken into small fragments related to the weight loss of the neat PHBV. After three months of being buried in the soil, the mass loss of PHBV was approximately 37%. Interestingly, the biodegradability of the blends was more pronounced, showing a mass reduction of approximately 40% and 45% for ENR-25 and ENR-50, respectively. This could be caused by the poor compatibility level of the blends^22^ and fragmented into smaller phases compared to the neat PHBV and the blends with 5wt% PB-g-MA due to the presence of PB-g-MA improved the compatibility between PHBV and ENR phases.

## Conclusions

Epoxidized natural rubbers (ENRs) were effectively used to improve mechanical performance, particularly the toughness of PHBV. By melt blending process, the varying epoxide concentration of ENR (ENR-25 and ENR-50) has no significant influence on improving the impact property of the blends, while adding 5wt% polybutadiene grafted maleic anhydride (PB-*g*-MA) as a compatibilizer significantly affected. In this study, the favorable blending ratio delivering the highest toughness was 70/30 PHBV/ENR. The toughness of the blends with 5wt% PB-*g*-MA incorporation was approximately 200% greater than that of the neat PHBV. Furthermore, the biodegradability of PHBV/ENR blends was more substantial than that of neat PHBV, showing a mass reduction of approximately 40% and 45% for the blends ENR-25 and ENR-50, respectively. However, the mass loss of PHBV was approximately 37% after 3 months of soil burial. Consequently, the developed PHBV/ENR blends showed good properties for potential use as biodegradable packaging. Thus, using ENRs, derived from renewable resources for toughening PHBV could pave the way for broadening and diversifying ENRs and PHBV for eco-friendly and sustainable purposes.

## Materials and methods

### Materials

Poly(3-hydroxybutyrate-co-3-hydroxyvalerate) (PHBV, ENMAT Y1000P) was purchased from Ningbo Tianan Biologic Materials Co., Ltd., China. Epoxidized natural rubber with 25 and 50% epoxidation contents (ENR-25 and ENR-50) was purchased from Muang Mai Guthrie Pub Co., Ltd., Thailand. Polybutadiene grafted with maleic anhydride (PB-g-MA, Ricobond® 1756) was manufactured by Cray Valley LLC, USA. The PB-g-MA has a low molecular weight (2500) with the grafting of 3 MA groups per chain. The epoxidation natural rubber was used as received. However, PHBV pellets were dried at 60 °C for 24 h before usage, and PB-g-MA was stored in a desiccator to prevent moisture.

### Preparation of PHBV/ENR blends

PHBV/ENR blends were prepared via the melt blending method using an internal mixer. The dried PHBV was first added in an internal mixer at a temperature of 175 °C with 50 rpm of rotating speed for 5 min to soften PHBV. Then, ENR and PB-g-MA were added, and the mixing was continued for another 5 min under the same conditions. The weight ratios of the PHBV to ENRs (ENR-25 and ENR-50) were presented as follows: 100/0, 90/10, 80/20, and 70/30, with 5 and 10 wt% of PB-g-MA. The obtained PHBV/ENR blends were milled. Then, the standard test samples were formed using a compression molding machine operated at 180 °C.

### Thermogravimetric analysis (TGA)

The thermal stability of the PHBV pellet (unprocessed), ENR-25, ENR-50, and the PHBV/ENR blends with and without PB-g-MA were examined using STA 2500 (Netzsch, Germany). The samples were heated from room temperature to 600 °C at a heating rate of 10 °C min^-1^ under N_2_ atmosphere, and their thermal degradation temperatures (T_d_) were determined.

### Differential scanning calorimetry analysis (DSC)

The thermal transitions of PHBV/ENR blends were investigated using 3500 Sirius (Netzsch, Germany). The samples were first heated from room temperature to 200 °C at a rate of 10 °C min^-1^ under N_2_ atmosphere and subsequently cooled to − 70 °C at a cooling rate of 10 °C min^-1^, then reheated from − 70 to 200 °C with the same conditions and cool down into room temperature. The glass transition temperature (T_g_), crystallization temperature (T_c_), melting temperature (T_m_), enthalpy of fusion (ΔH_m_), and degree of crystallinity (X_c_) were reported.

### X-ray diffraction (XRD)

XRD analysis of the neat PHBV and PHBV/ENR blends with and without PB-g-MA was performed using an X-ray diffractometer (D8 advance Bruker, USA). The XRD patterns were recorded in the range of 5–80°. The Sherrer’s equation is used to calculate and determine the crystal size by D = Kλ β^-1^cos(θ)^-1^, where D is the crystal size, K is the Scherrer’s constant (0.9), λ is the wavelength of X-ray, β is full width half maximum; FWHM, θ is the scattering angle^58^.

### Mechanical characterization

Tensile testing was conducted according to ASTM D638 using standard dumbbell-shaped samples (type 4) with the dimensions of 6 × 115 × 3 mm^3^ and a grip distance of 115 mm. The tensile testing was performed under the load cell capacity of 5 kN and a crosshead speed of 50 mm min^-1^. Furthermore, the flexural testing was conducted according to ASTM D790 standard. The rectangle-shaped samples with the dimensions of 12.7 × 127 × 3 mm^3^ were tested under the load cell capacity of 5 kN and a crosshead speed of 2 mm min^-1^. The tensile and flexural tests were conducted using the universal testing machine Tinius Olsen H50KS (Calserve, Thailand). Moreover, the notched Izod impact testing was done according to ASTM D256 standard using the notched impact samples dimensions of 12.7 × 63 × 3 mm^3^ with a 22.5° notched. All samples were notched using a notching machine (Taiwan Shang Yang, Taiwan), and the impact test was performed using an impact tester (Gotech, Taiwan). The standard test specimens for tensile testing, flexural testing, and impact testing were prepared via the compression molding process. The reported average and standard deviation (SD) values were calculated from at least five samples.

### Statistical analysis

Statistical significance for the mechanical properties was determined using SPSS software (version 22.0; SPSS, Inc., Chicago, IL) using a one-way ANOVA with Duncan’s multiple range post-hoc test. All data were obtained from triplicate measurements and presented as the mean ± SD.; *P* values < 0.05 were considered to be statistically significant.

### Scanning electron microscopy (SEM)

Blend morphology was examined via the fractured samples obtained from the impact testing using a scanning electron microscope (JSM – 6480LV, Jeol, Japan). Before being characterized, all samples were coated with gold using an SPI module sputter coater (Structure Probe, Inc., USA). Then, the SEM images were obtained with a voltage of 15 kV. Furthermore, the blend morphology evolution after biodegradation was also investigated. The samples obtained from the soil burial test were coated with gold, and the SEM images of the samples’ surfaces were obtained with a voltage of 15 kV.

### Biodegradability

For the biodegradability test, the 70/30 PHBV/ENR-50 blends with and without 5wt% of PB-g-MA and neat PHBV were selected as the most promising toughened blend composition and reference sample, respectively. According to the soil burial testing procedure, the samples were buried below the ground for 20 cm for 3 months^59,60^. The burial test was performed in Bangkok, Thailand. At the desired time of 1, 2, and 3 months, the soil humidity is maintained at approximately 40%. After being buried at the desired time, the samples were subsequently taken off the soil, washed in distilled water, and dried in an oven at 60 °C for 24 h. Then the samples were kept in a desiccator to prevent moisture.

## Data Availability

The datasets used and/or analyzed during the current study are available from the corresponding author on reasonable request.
